# Motor related potential amplitude and latency shifts with repetitive task execution as an indication of motor adaptation

**DOI:** 10.3389/fnhum.2026.1845451

**Published:** 2026-07-30

**Authors:** Julian A. Martinez, Ghazala T. Saleem, Sue Ann Sisto, Albert H. Titus, Filip Stefanovic

**Affiliations:** 1 Department of Biomedical Engineering, University at Buffalo, Buffalo, NY, United States; 2 Department of Rehabilitation Science, University at Buffalo, Buffalo, NY, United States

**Keywords:** event related potentials, motor related cortical potential, motor related potentials, pre-motor cortical activation, readiness potential, repetitive motor task, short-term motor learning, task modulation

## Abstract

Motor Related Potentials (MRPs) measure cortical activity relative to a motion task. We evaluated MRPs for a repetitive wrist flexion/extension task to identify changes in the signal characteristics that are indicative of task performance and motor adaptation. Our experiment consisted of a custom-built isometric joystick that controls a cursor on screen, while EEG and EMG data are recorded. The participants (6 healthy adults) performed more than 60 task repetitions, including flexion, extension, and alternating wrist movements while using the joystick. Task performance was tracked as successes or failures based on the individual’s ability to move the cursor to a target and hold the position. Our results show that MRP latency changes gradually with consecutive successful tasks (13–35%), and changes more abruptly (random) with failed tasks. Similarly, failed tasks see, on average, a 133.6 ms longer latency than successful tasks. Finally, statistical evaluation of MRP features identified three significant characteristics that can be used to distinguish between successful and failed tasks (Max Amplitude, Min Amplitude, P–P Amplitude). This research suggests that MRPs change with motor adaptation and performance for volitional reaching tasks. We propose that long-term applications of this research include measuring learning rates and performance outcome predictions for healthy and impaired motor control assessment.

## 1 Introduction

Cortical activation acts as a measurable initialization of brain processing and movement execution. Changes in cortical activation are clinically significant as there are notable differences in any diseased states such as Parkinson’s Disease, Cerebral Palsy, stroke, and traumatic brain injury (Kiper et al., 2016). However, little is known about these cortical manifestations and how they change over time based on motor control strategy and learning. Electroencephalography (EEG) is a common tool used to explore pre-motor planning, preparation, and motor execution through the analysis of motion related potentials (MRP) and frequency spectral power changes (Colebatch, 2007; Kirsch et al., 2010). These potentials are a subset of event-related potentials (ERPs) that measure the causal relationship between brain activity and motor outcomes. In this study, we explore ERPs during repetitive volitional motion tasks to quantify how they change due to motor learning, and identify differences when tasks are complete successfully or not. We hypothesize that these changes in MRPs are indicative of short-term adaptation and can support data-driven techniques to classify successful movement control for assistive devices.

Earlier studies have shown that MRPs appear in sequential phases 1 to 2 s prior to motion onset, and 0.5 to 1 s after motion onset. The initial MRP phase is defined by a slow negative slope in the EEG signal, known as the Bereitschaftspotential (BP) or readiness potential (RP)(De Bruijn et al., 2003; Colebatch, 2007; Kirsch et al., 2010). The second phase is a steeper negative slope that occurs closer to the motion onset (~500 ms prior) known as BP2 or the late BP(Colebatch, 2007). This is then followed by two additional characteristics that are considered more variable: (1) the pre-motion positivity (PMP) which occurs between the late BP and motor potential; and (2) the laterization motor potential (MP) which can vary in amplitude, duration and latency, directly prior to motion onset (~50 ms) (Bortoletto et al., 2006). These characteristics serve as benchmarks for relative analysis in the MRPs, allowing changes in cortical activation to be evaluated with pre-defined guideposts. Specifically, earlier studies have shown that the MRP is often spatially related to motor context and task sub-types. For example, the supplementary motor area (SMA) is largely responsible for early-BP with greater activation than the motor cortex for hand movements (Shibasaki and Hallett, 2006). While the late-BP and motor potential (MP) have their greatest activation in the contralateral motor cortex, suggesting that BP activation becomes apparent over the M1 region just prior to motion onset contralaterally (Shibasaki and Hallett, 2006).

Regardless, the components of MRPs have demonstrated significant dynamics across healthy populations. For example, movement complexity is known to produce a greater BP when compared to that of simpler motor tasks (Slobounov and Ray, 1998). In addition, it has been found that the amplitude and onset of MRP are affected by pre-motor prompts for motion tasks. For example, greater amplitude changes between early-BP and late-BP can be seen in self-initiated movements when compared to regularly cued movements, as well as increased MP amplitudes (Jankelowitz and Colebatch, 2002). Whereas regularly cued movements showed earlier onset of both early-BP and late-BP as participants began predicting motion onset – also a form of response due to learning (Jankelowitz and Colebatch, 2002). Other factors such as force exertion, speed of motion (Rapid vs. Slow increases in force), precision, and discreteness of the motion have also shown to have effects on the characteristics of MRPs(Shibasaki and Hallett, 2006). For example, motions requiring greater or faster forces show increased negativity of BP and occur closer to contraction onset (De Bruijn et al., 2003; Shibasaki and Hallett, 2006).

Interestingly, clinical studies of MRPs in people with neurological disorders have shown that different diseased states affect the MRP characteristics(Shibasaki and Hallett, 2006; Colebatch, 2007). For example, in the case of brain lesions, there is decreased MRP amplitudes in the regions where the lesions occurred – e.g., cerebellar lesions are reported to have the largest attenuation due to the cerebellar projection to the motor cortex (Shibasaki and Hallett, 2006, Colebatch, 2007). Similarly, stroke-induced hemiparesis has been reported as a loss of laterality of late BP which shifts the MRP component (Shibasaki and Hallett, 2006, Colebatch, 2007). Jankelowitz and Colebatch expanded on these findings by exploring MRPs related to acute weakness in subcortical stroke patients and found that the greatest change occurred in the peak motor potentials prior to motion onset (Jankelowitz and Colebatch, 2005). However, these studies showed high variability due to the experiment design, preparation, and level of intention (i.e., self-initiated vs. cued, max effort vs. low effort). Regardless, it is still suggested that MRP features could measure changes due to motor learning, short-term adaptation and cortical modulation (Smith and Staines, 2010).

Additional studies into healthy populations have shown that other factors have effects on MRP activity. For example, Freude and Ullsperger’s experiments with fatiguing and non-7dfatiguing hand movements found that BP amplitudes were dependent on force level (Freude and Ullsperger, 1987). Furthermore, it was found that high-force fatiguing contractions (80% Max Voluntary Contraction (MVC)) caused MRP amplitudes to double at Cz and quadruple at the lateral motor cortex electrodes (C3, C4) (Freude and Ullsperger, 1987; Lattari et al., 2014). They also found that with repetitive movements at 20% MVC there was also an increase in BP which could be caused by a higher intention to modulate the lower force level. Another study conducted by Dirnberger and colleagues dissociated motor preparation from memory and attentional processes using MRPs (Dirnberger et al., 2000). There, they found that regular repetitive tasks of the same movement had less activity than random repetitive tasks with different movements. To determine if the increase was due to motor preparation or memory/attentional processing they studied a third condition which was an alternating task between two movements, giving it the predictability of the regular task, but with the change in movement of the random task. With this third task they found that there was a similar increase in activation than that of the random task over the motor areas, suggesting that the increase in activation was a result of increased motor preparation (Dirnberger et al., 2000). Furthermore, they found that the random task had larger activation in the contra-lateral frontal regions which could be associated with the memory/attentional processing needed to randomize the movements (Dirnberger et al., 2000).

Ultimately, this research suggests that the MRPs are dynamic based on the movement context, and that they have spatial and temporal patterns that correspond to movement context. In this study, we aim to demonstrate that temporal properties of the MRPs not only change with task repetition, but that they are also dependent on successful completion of the task. In other words, learning to perform a task affects the MRP characteristics differently than failing to achieve the task. In this study, we measure the changes to MRPs in healthy individuals performing a repetitive voluntary motor task with a volitional task. We aimed to identify key features of movement-related cortical activity, and to identify changes that occur due to motor adaptation. As such, we have two primary hypotheses: (1) Successful and failed task execution attempts will produce different characteristic MRPs measured by peak amplitude and peak latency in motor planning and motor execution; (2) Longitudinal changes in MRPs with task completion are due to motor adaptation.

By exploring these hypotheses, we aim to demonstrate that modulatory changes in the corticomuscular system manifest through task repetition in the MRP dynamics. Additionally, we will identify cortical activation profiles and key features related to task performance. This study is part of a larger project that aims to quantify neurorehabilitation from a neurological perspective, and to provide more objective metrics to clinicians for assistive technologies to support recovery.

## 2 Materials and methods

### 2.1 Participants

For this study, data were collected from six individuals (Female n = 4, Male n = 2) between the ages of 18–40 years old. All participants provided voluntary consent to participate in the study and were informed of the possible side effects of the study, along with the inclusion criteria of being a healthy individual with no underlying health conditions. While exclusion criteria included any contraindications to functional magnetic imaging (fMRI), transcranial magnetic stimulation (TMS), or transcranial direct current stimulation (tDCS)-i.e. implanted medical devices, metallic objects in the body, history of seizure and/or unexplained fainting, as well as any history of neurological or non-neurological conditions. Informed consent was collected from all participants prior to data collection and participants were compensated upon completion of data collection.

### 2.2 Experimental procedures

**Figure 1:**
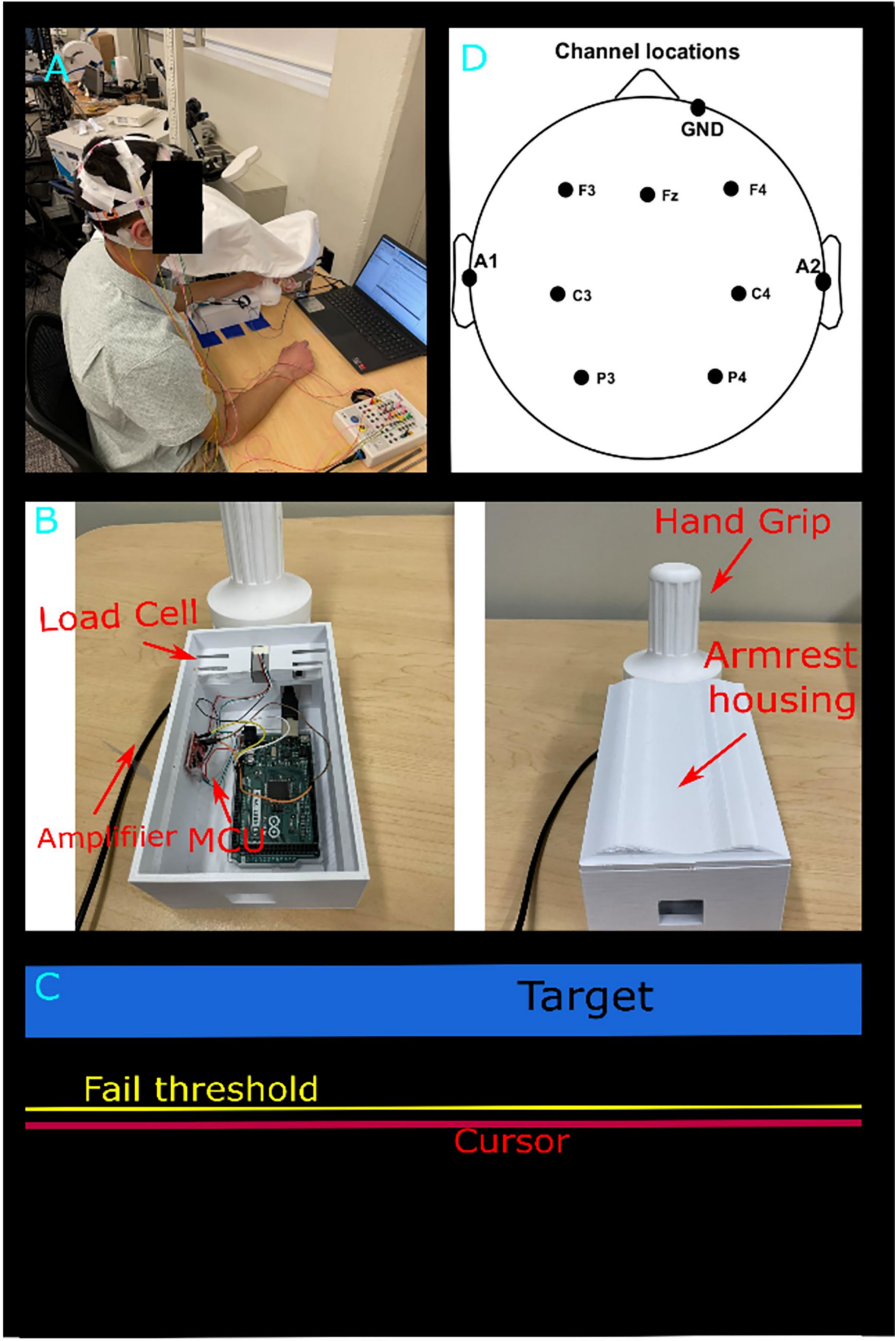
Experimental setup. **(A)** Image of participant seated in position gripping the HMI and sensors placed on body; **(B)** HMI components and complete look; **(C)** GUI developed in Psychtoolbox to move a cursor on screen to a target; and **(D)** EEG channel locations.

The participants performed a repetitive motor task comprised of wrist flexion and extension while gripping an isometric joystick with their non-dominant hand. They were instructed to exert forces on the joystick to move a cursor on screen to a target area presented on a custom-built graphics user interface (GUI) (Figure 1A). The isometric joystick – or human-machine interface (HMI) – was built in-house using an Arduino Mega R2 (Arduino, Italy), a straight bar load cell (SparkFun, USA), and a Hx711 load cell amplifier (SparkFun, USA) (Figure 1B). The Arduino was connected to a PC via cable, and the GUI was designed in PsychToolbox (Kleiner et al., 2007); displayed on an 18in (1920×1080), 60 Hz monitor (Figure 1C). The device was programmed such that wrist flexion would move the cursor down (negative pixel movement) while wrist extension would move the cursor up (positive pixel movement).

The experiment consisted of 60 target motions which were broken into three 20-task blocks. The tasks blocks consisted of 20 flexion tests, 20 extension tests, and 20 alternating flexion and extension tests (10 of each) with 10 s of rest between blocks and 2 s of rest between tasks. During each task, a straight blue bar target was presented on the screen (Figure 1A). The participants would then move their cursor (thin red bar) to the target and then hold the position for 2 s. A threshold line (yellow line) was depicted halfway between the starting position and the target to prevent participants from returning to rest prior to test completion. If the cursor crossed the threshold twice, the software would score the task as a failure, then present a reset screen, and then restart the task. Upon successful completion of the task, a rest screen would appear indicating the time until the next test (Figure 1A). The success/fail rates were then calculated (Equations 1, 2).


$$SuccessRate=\frac{\#successfulattempts}{\#totalattempts}x100$$ (1)

$$FailRate=\frac{\#failedattempts}{\#totalattempts}x100$$ (2)

During setup, electromyography (EMG) electrodes (Delsys Trigno, USA) were placed on the Flexor Capri Radialis (FCR) and the Extensor Capri Radialis (ECR), to collect muscle activity during the task. The recording electrodes were placed over the muscle belly using motor points and palpation. The reference electrodes were placed on the bony portion of the posterior elbow. Additionally, brain activity was recorded using a Mitsar 202 EEG amplifier and standard 10–20 electrode montage. We defined the primary recording locations as Frontal electrodes (F3, Fz, F4), Central Electrodes (C3, C4), and Parietal electrodes (P3, P4) (Figure 1D), with reference electrodes placed at A1/A2 and references placed on the temple to achieve low impedances (< 15 kOhm). All data were collected at a sampling rate of 2 kHz with an integrated notch filter at 60 Hz applied to the EEG. Digital root-mean-square (RMS) enveloped EMG data were processed for both muscles. The EMG, EEG, and cursor positions were recorded and time-synchronized using the internal clock timestamps and trigger. Additional study protocol details can be found here (Stefanovic et al., 2023).

### 2.3 Data processing

All data processing was performed in MATLAB. The data were time-synchronized and segmented into 5 data blocks: Calibration, Baseline (BL), Flexion Task (FT), Extension Task (ET), and Alternating Tasks (AT).

#### 2.3.1 EMG data

The raw EMG data was bandpass filtered from 10 to 400 Hz with 4th order Butterworth filter. The signal was then rectified, and the RMS envelope was extracted. The envelope was then used to validate the RMS channel calculated by the sensors during recording while a 60s sliding window function was used to remove baseline drift. Contractions were labeled using EMG peaks that were 3 times the standard deviation of the baseline activity. The contraction durations, peaks and average amplitudes were calculated using these labels.

#### 2.3.2 EEG data

**Figure 2:**
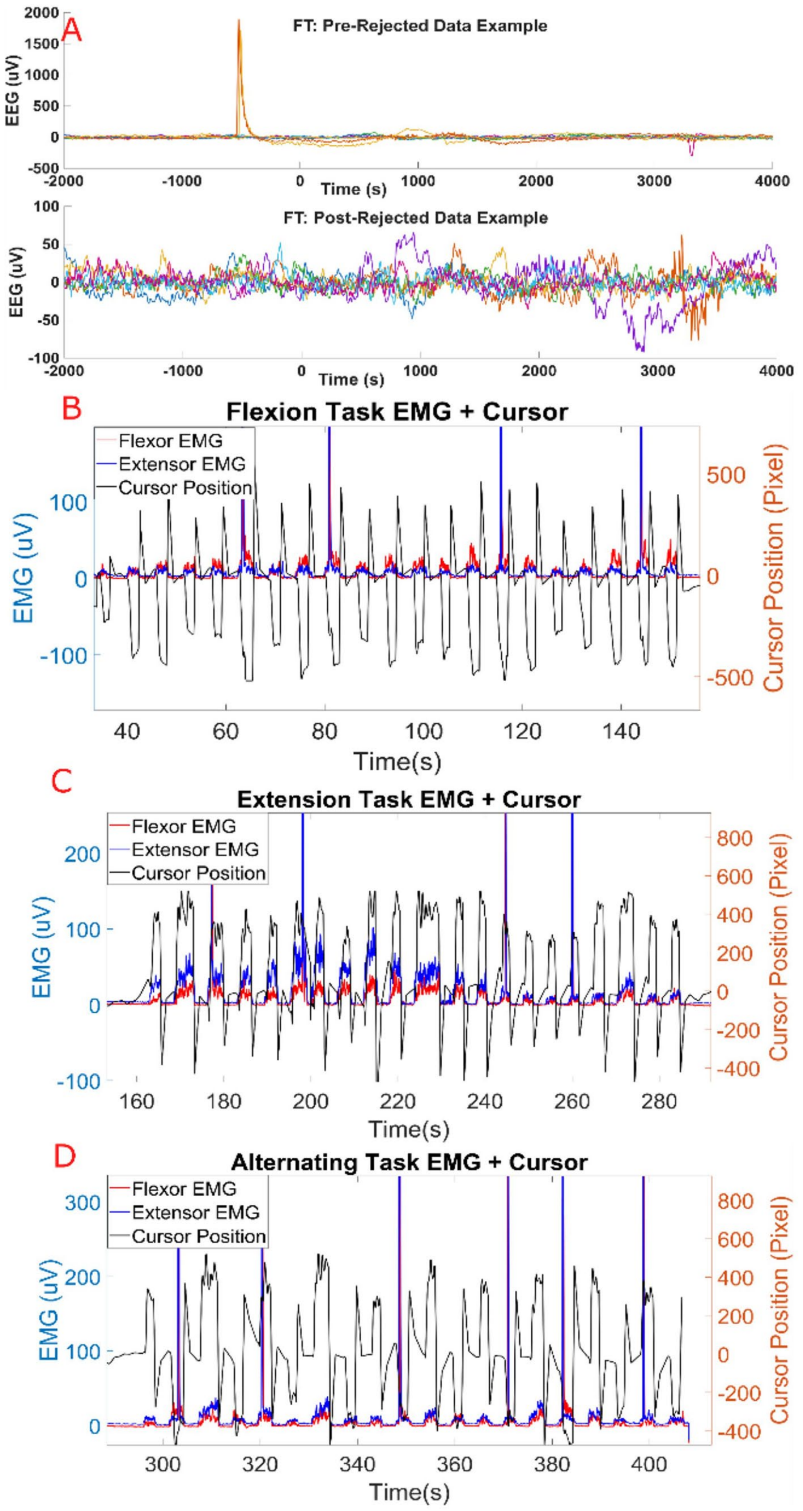
**(A)** Data comparison of epochs pre- and post- threshold rejection. The top plot a large artifact in epoched data and the bottom plot shows the data after rejecting the bad epochs. **(B–D)** Experimental data showing time-synched EMG and cursor data.

The EEG data were processed using the EEGLab toolbox in MATLAB. The toolbox uses event labels to format the data for use. Here event labels were used to signify the start of experimental blocks, Successful contraction onset/offset, and failed contraction onset/offset. The data were then imported into the toolbox where the channel locations were defined, and the event markers were extracted. The data were then filtered using the built-in bandpass filter (0.5 Hz to 90 Hz) to remove unwanted noise, and the notch filter was applied at 60 Hz ± 2 Hz to attenuate any powerline noise. Using the epoch extraction tool, the data were then segmented into datasets (1 EEG file produced 2 epoched datasets). The epochs were defined by the event markers representing contraction onset for successful and failed attempts. These data sets were then inspected for any epoch that contains artifacts (e.g., Blinking, Body or Head Motion, Stimulation). A threshold of ± 200 μV was used to remove any epoch containing contaminated data––see Figure 2A. Additionally, independent component analysis (ICA) was applied to the data sets and components were labeled using the ICLabel tool. Components containing >80% artifacts were rejected and the data were reconstructed after component removal. Following removal, the Event Related Potentials (ERP) and Slow-Wave MRP analysis were completed.

#### 2.3.3 Extracting ERPs and slow-wave MRPs

Event related potentials were calculated using the precomputation function in EEGLab. This uses the average EEG activity for a given channel across all the defined epochs. In this case, the successful task ERPs are calculated as the average of the uncontaminated successful tasks for each individual EEG channel. The same process is repeated with the failed task dataset. Lastly, a grand average across the group of subjects is computed for success and fails for comparison. Then, five consecutive epochs are averaged together to calculate ERPs relative to task duration using the ERPimage function (Hong et al., 2009; Makeig and Onton, 2011; Delorme et al., 2015). Thus, we produce multiple ERPs for each subject to evaluate the ERPs from multiple perspectives (Event-Latency and Repetitions).

For the evaluation of the slow-wave MRPs, the processed ERP data was low-passed filtered using a 350th-order median filter using the following equation(Pratt, 2007; Jankelowitz and Colebatch, 2002):


$$y\left(k\right)=med[x\left(k-\left(\frac{n}{2\;}\right):k+\left(\frac{n}{2}\right)-1\right]$$

**Figure 3:**
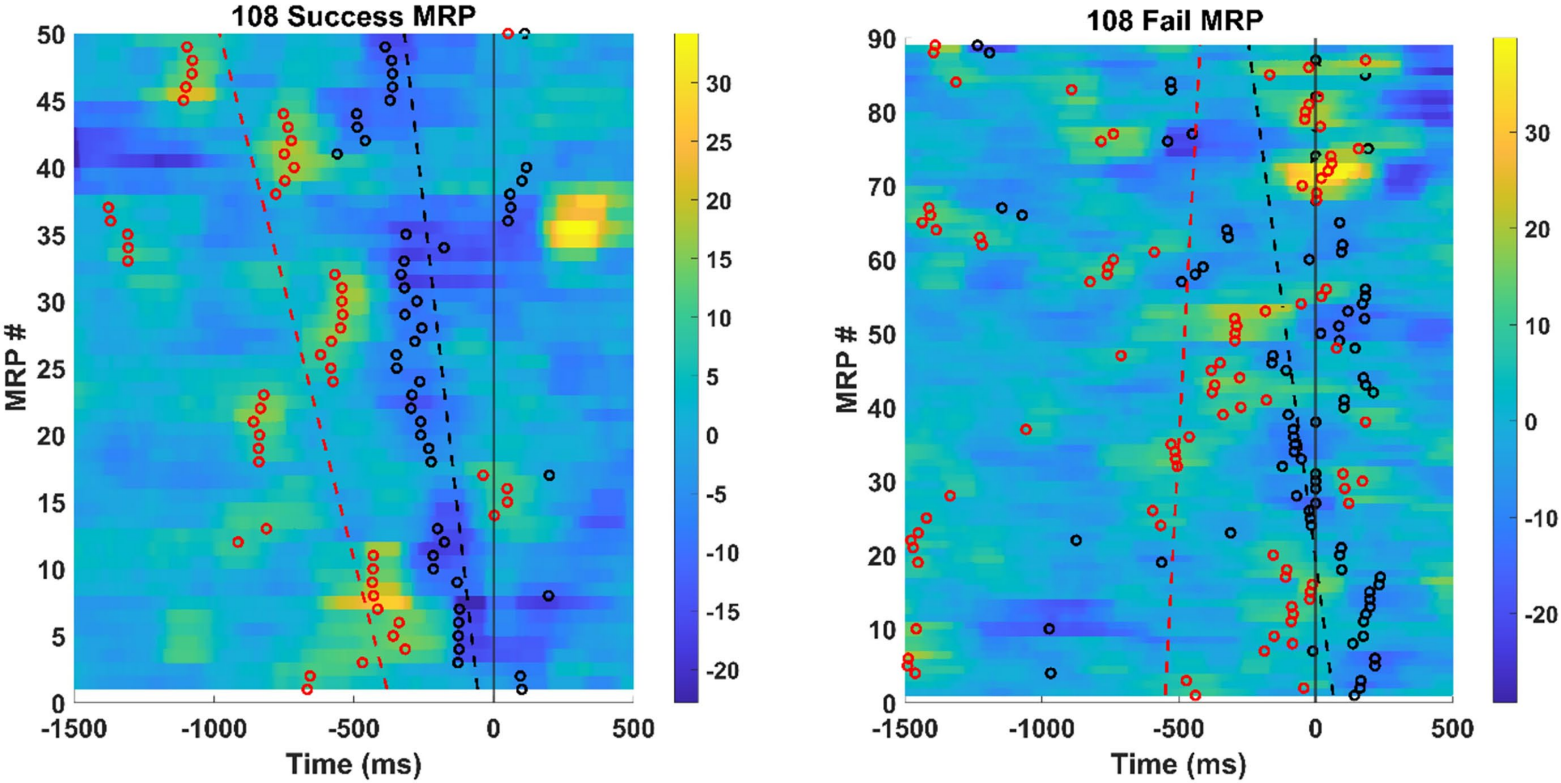
Representative slow-wave plots at C4 for MRPs for Subject108. Plots are shown for −1,500 ms to 500 ms after contraction onset (Left-Success MRPs, Right-Fail MRPs). Red markers label peak amplitude latency, and black markers mark trough latency. The dashed black lines represent approximation of trough latency movement for visualization, while dashed red lines representing peak latency movement.

Where y is the filtered data, x is the ERP data, k is the length of the median data (12000), and n is the order of the filter (350). This data was then plotted as 2D-surface plots, with MRPs plotted along the *y*- axis, MRP time along the *x*-axis, MRP amplitude represented by the color [yellow (+), blue (−)], shown in Figure 3. Features were extracted from the individual MRPs using the *findpeaks* functions from -1500 ms before contraction onset to 250 ms after contraction onset with a minimum distance between peaks of 25 ms. Extracted peaks were plotted as red markers and troughs as black markers. Features latency changes across tasks were fit using the *polyfit* function. Lines of best fit were plotted as red dashed lines for peaks and black dashed lines for troughs.

#### 2.3.4 Statistical analysis

To determine the significance of the features extracted from the MRP, a linear mix-effect model (LME) was applied to the data for analysis in MATLAB. From the MRP data, 10 features were extracted for analysis, and model parameters were defined using syntax: ′feature~conds∗trials+(1|sub)′.

The fixed effects are defined by the term *conds*, which represents Boolean performance classification (success/fail), while the term *trials* represents the attempt # (i.e., Success1, …, SuccessN or Fail1, …, FailN). The subject is used as a random effect. Additionally, the term ‘conds*trials’ tests the significance of the interaction between task performance and trials. Then, the Benjamini and Hochberg false discovery rate (FDR) correction was applied to ensure robustness of the analysis. Additional models were tested using random slope terms in which the (1|sub) term was replaced with (1 + trials|sub) or (1 + conds|subs). This models the data under the assumption that each subject has their own unique response, or performance, between attempts and acts as a sensitivity test for feature significance.

## 3 Results

### 3.1 EMG and EEG data

The processed and synchronized data are shown in Figure 2. where, flexor (red) and extensor (blue) muscle EMGs are shown alongside the cursor data (black) collected from the HMI. Data are provided for all three blocks, including the flexion task (Figure 2B), extension task (Figure 2C), and alternating task (Figure 2D). The flexion and extension tasks demonstrate more homogeneous dynamics, while the alternating task exhibits more variability (i.e., heterogeneous dynamics) across the EMGs and cursor movements. The peak height of the cursor indicates how far the cursor moved from the start to reach the target, while the amplitude of the EMG indicates the strength of the contraction. The EEG data can be corrupted by spurious activity (Figure 2A, top panel) such as motion artifacts, and other events. Thus, Figure 2A, bottom panel, demonstrates the effect of epoch-based removals to minimize skewed data.

### 3.2 Task performance metrics

Table 1 displays the proportion of successful and failed task attempts per participant. The flexion task (FT) accounted for the most task attempts with failures out of the 3 exercise blocks, followed by the extension task (ET) and alternating task (AT), respectively. On average there were 6.33 ± 4.23 tasks with at least one failure FT, 5 ± 4.69 during ET and 4.83 ± 3.19 during AT. Recall, that a single task can be failed more than once. Furthermore, Table 1 shows that subject S105 had the greatest number of repeated task failures with 354 total failed attempts out of a total 414 trials (85%). Across the group, the average failed attempts were 95.83 ± 131.18 attempts out of an average of 155.83 attempts (61.5%). When removing S105 from this, the average failed attempts were 44.2 ± 38.9 (28.3%).

**Table 1:** Subject success/fail performance.

Sub	FT failed (×/20)	ET failed (×/20)	AT failed (×/20)	Total failed attempts	NMES fails	Total success rate (%)	Total time (s)	Time per task (total time (s)/60 tasks)
S102	6	12	9	55	10	52.2	821.85	13.70
S104	6	3	4**	17	4	77.9	512.70	8.55
S105	13	9	6*	354	10	14.5	3632.17	60.54
S108	8	5	7*	102	5	37.0	892.10	14.87
S112	5	0	3*	46	1	56.6	641.81	10.70
S114	0	1	0	1	1	98.4	410.61	6.84

NMES fails are failures that occur during perturbations, *All flexion tests, **3 out of 4 flexion tests with 2 NMES Fails.

Table 1 demonstrates that subjects S104 and S114 had the highest success rates (78 and 98% respectively), while S105 and S108 had the lowest (14 and 37% respectively). Also, S102 and S112 both showed moderate success with a rate of 52.2 and 56.6%, respectively. Across all participants the average success rate was 56.1% with a variance of 8.7%. The average time per task was 10.9 s, with a variance of 11.4 s when excluding S105.

### 3.3 Task performance and ERP

**Figure 4:**
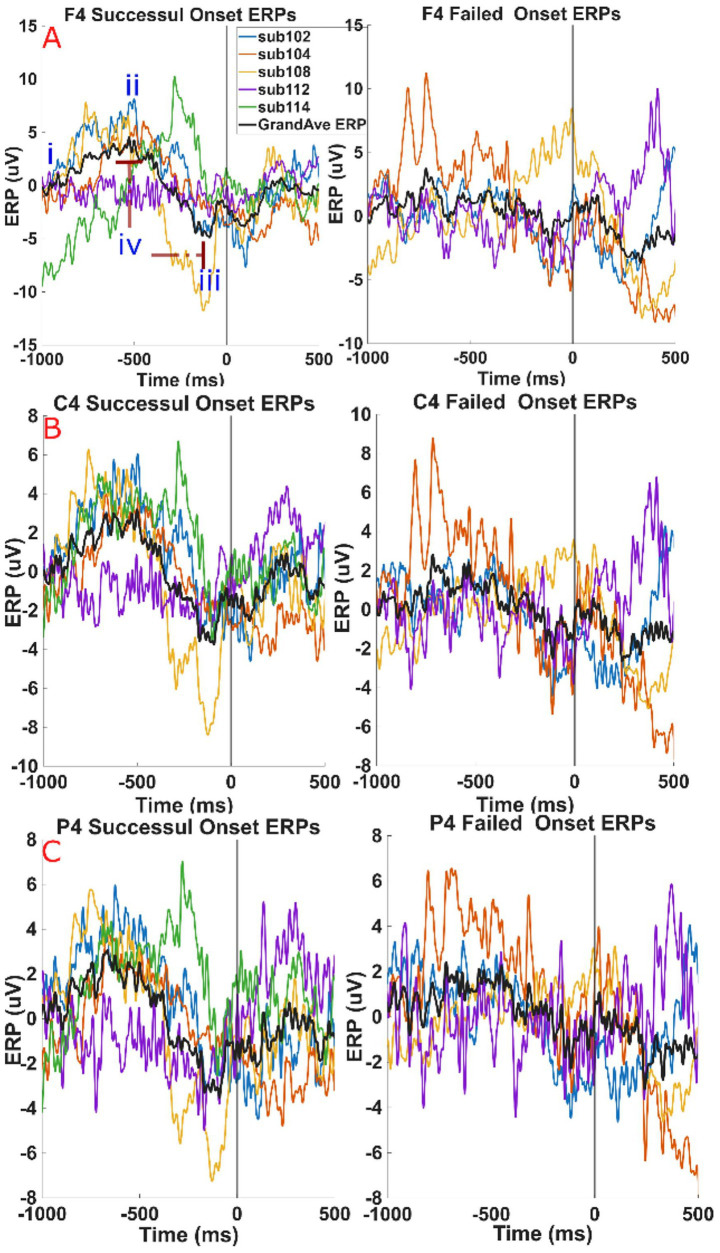
Task success/fail averaged motor related potentials (MRPs) for all participants. Data are shown for F4, C4, P4 **(A–C)** (Left) MRPs for successful tasks; (Right) MRPs for failed tasks. Color coded (Sub102, Sub104, Sub108, Sub112, Sub114) and the black line representing the grand average. Muscle contraction occurs at *t* = 0, while *t* < 0 represents EEG activity prior to motion onset. In **(A)**: (i) Pre-peak trough, (ii) Peak amplitude, (iii) Trough, (iv) Peak-to-peak amplitude.

Figure 4 shows the difference in measured MRP for successful (left side) and failed (right side) tasks. Each task MRP is overlayed on the image for all participants, with the colored lines representing the average MRP across all tests for each subject, while the black line represents the grand average MRP across all subjects for that given channel. Here we show three channels (namely F4, C4, and P4) as they were considered representative. In the successful tasks an increase in cortical activation was observed as an envelope between 200 and 800 ms prior to contraction onset (occurring at t = 0) and was followed by a decrease in activation relative to baseline. Here, the grand average across the group for the maximum MRP amplitude was 3.63 ± 0.79 μV for successful test. The associated average latency was 547 ± 80 ms. On the other hand, the failed task ERPs had a maximum average amplitude of 3.24 ± 0.65 μV and a latency of 680 ± 92 ms across the group. The detailed MRP amplitude and time ranges are displayed in Table 2.

**Table 2:** Peak MRP amplitude and time of occurrence.

	Average maximum success MRPs			Average maximum fail MRP		
Sub	Amp (μV)	Lat (ms)	P–P (μV)	Amp (μV)	Lat (ms)	P–P (μV)
S102	6.94 ± 1.31	558.9 ± 112.3	11.23 ± 2.38	3.47 ± 0.44	777.4 ± 183.7	8.56 ± 0.87
S104	5.00 ± 1.26	572.6 ± 85.8	8.37 ± 1.82	9.68 ± 2.22	735.9 ± 46.4	15.37 ± 4.07
S108	6.69 ± 1.09	671.5 ± 96.4	15.11 ± 3.12	5.37 ± 2.87	730.7 ± 184.3	8.49 ± 4.00
S112	2.25 ± 0.79	307.4 ± 443	6.06 ± 1.25	3.28 ± 0.95	658.3 ± 370.1	7.19 ± 0.74
S114	8.42 ± 1.70	281.9 ± 1.4	13.4 ± 4.00	N/A	N/A	N/A
AVG	3.63 ± 0.79	546.9 ± 80.5	7.16 ± 1.56	3.24 ± 0.65	680.6 ± 91.5	5.91 ± 1.07

In Tables 2, 3, the EEG channels (F3–P4) are listed on the left, while MRP amplitudes (Amp), latencies (Lat) and peak-to-peak (P–P) are shown. Peak-to-peak voltages are defined as the voltage from peak to trough in the MRP prior to motion onset. Voltages are provided in microvolts, while latency is given in ms. Latency is defined as the time prior to motion onset – thus a larger time indicates greater latency between the peak and motor onset. The data are split to show differences in the MRP characteristics across successful and failed tasks. Averages are also provided.

**Table 3:** Channel location average peak MRP amplitude and latency.

	Average maximum success MRPs			Average maximum fail MRP		
Chan	Amp (μV)	Lat (ms)	P–P (μV)	Amp (μV)	Lat (ms)	P–P (μV)
F3	6.76 ± 2.42	381.1 ± 211.3	12.67 ± 4.15	6.55 ± 4.09	605.6 ± 420.8	12.12 ± 5.36
Fz	7.28 ± 3.29	370.8 ± 207.9	12.99 ± 5.33	6.64 ± 4.62	669.1 ± 448.7	12.07 ± 4.91
F4	6.72 ± 3.35	580.9 ± 252.9	13.14 ± 6.44	6.46 ± 4.08	634.4 ± 430.6	11.52 ± 4.52
C3	5.57 ± 1.68	420 ± 254.7	9.81 ± 2.22	5.17 ± 3.41	432 ± 414.2	9.50 ± 5.92
C4	4.86 ± 2.25	451.2 ± 286.9	9.20 ± 3.78	4.38 ± 2.99	415.4 ± 301.8	8.60 ± 3.72
P3	4.86 ± 1.79	481.5 ± 309.6	8.84 ± 2.31	4.67 ± 1.88	554.9 ± 301.8	7.60 ± 2.55
P4	4.97 ± 1.93	663.2258.7	9.13 ± 2.51	4.29 ± 1.59	616.3 ± 417.9	7.9 ± 1.92

When evaluating the ERPs with respect to task performance we can see that there is a difference between the average ERP latency across successful and failed attempts for all subjects. For example, the latency is longer across all failed tasks, such that the peak typically occurs 133.64 ms later than successful tasks. For individual EEG channels (Table 3), the same trend appears. Furthermore, the latency for the frontal channels (F3, Fz) is shorter than the central/parietal regions (C3/C4, P3/P4). For example, peaks for F3 and Fz occurred 381.1 and 370.8 ms prior to contraction onset, the F4 peak occurred at 580.9 ms prior to onset in the successful MPRs, while C3, C4, P3, P4 peaks occurred between 663.2 and 420 ms prior to contraction onset. In failed tasks, the peaks for the frontal channels occur between 670 and 600 ms prior to motion onset. Additionally, the failed ERPs showed greater variability in latency. In the frontal channels there was a 70–116% increase in standard deviation (SD) in the failed tasks, when compared to the successful tasks. In the central channels this change was 63%.

When evaluating the peak activity of the MRPs it was seen that there was decrease in peak amplitude in the posterior channels, when compared to the frontal channels. For example, Fz showed the highest amplitude of 7.28 ± 3.29 μV in successful MPRs (Table 3), while C4/P3/P4 showed the lowest. This trend was also seen in the failed MRPs, with the frontal regions showing greater activity than central and posterior regions. As such, this trend seems ubiquitous regardless of success or failure. However, there was relative decrease across all channels in the failed task when compared to the successful task. For example, the amplitude decrease was 3, 9, 4, 7, 10, 4, and 14% for F3, Fz, F4, C3, C4, P3, and P4, respectively.

### 3.4 Effect of task repetition on ERP

Table 4 shows the peak amplitude (Amp) and latencies (Lat) for the MRPs for all subjects across the three experimental blocks for successful tasks. Each block depicts multiple tasks, with amplitudes and latencies provided for each. MRP amplitude is provided in microvolts, while latency is given in milliseconds, where a time of zero indicates when motion onset occurs, while larger numbers indicate MRP peak times that occur before motion onset. In some blocks, MRPs were rejected as previously discussed and thus some blocks show less than 20 tasks per block. We color code the peak times to aid in visualization of peak time shift with task repetition. Specifically, the results show trends in MRP latency as the task is repeated – e.g., S104 starts with larger latencies (red) and moves to shorter latencies (green) during the FT block. Finally, participants data are depicted in the table except for subject S105 in Table 4 due to the number of failed attempts, and subject S114 in Table 3 due to a lack of failed task attempts. The data are depicted for only successful tasks in Table 4 due to the heterogeneity of the failed attempts across the group.

**Table 4:** Maximum ERP amplitudes (C4) for successful task.

Block		S102		S104		S108		S112		S114	
	Task	Amp (μV)	Lat (ms)	Amp	Lat	Amp	Lat	Amp	Lat	Amp	Lat
FT	1	12.4	574	19.5	1,034	14.8	606.5	11.0	38.5	19.0	279.5
	2	17.5	1,173	11.0	822	16.5	537.5	10.8	743.5	27.9	277.5
	3	21.4	1,173	10.7	827	18.1	1,084	12.3	743.5	23.4	274.5
	4	19.4	1,175	14.8	561.5	20.7	1,057	8.1	660	18.2	379.5
	5	16.7	1,175	20.9	560.5	18.0	284	6.5	674.5	17.4	729.5
	6	12.6	721.5	15.5	562	22.7	302.5	7.0	657.5	15.9	661.5
	7	12.2	627	19.2	490	34.2	344	7.2	102.5	13.5	428
	8	12.9	626	23.5	519	30.5	407.5	9.6	27.5	13.8	426
	9	13.9	625.5	19.8	539.5	28.4	407.5	9.3	28	10.1	823.5
	10	14.3	624.5	12.8	519.5	27.5	408	7.5	1,117	14.0	448
	…										
ET	1	16.4	387.5	11.7	668.5	17.2	670	11.3	344	15.2	818
	2	12.7	389.5	10.9	683.5	14.4	72	13.9	521.5	7.4	849.5
	3	10.4	928	14.1	670	18.3	850.5	12.4	523	10.0	117.5
	4	7.2	807.5	11.5	630.5	18.2	845	13.7	523.5	13.2	118
	5	9.1	806	15.4	631	21.3	847.5	11.3	615	7.9	118.5
	6	9.0	652.5	15.7	629.5	22.0	849.5	8.9	524	8.8	23.5
	7	10.1	397	15.5	629.5	20.2	780	7.9	1,045	15.3	253
	8	10.7	764.5	9.7	453	10.4	743.5	7.3	415	11.6	253
	9	10.7	398.5	11.4	566.5	12.7	608.5	6.8	499	9.8	251.5
	10	9.2	397	10.1	565.5	17.7	605.5	7.6	357	10.4	794.5
	…										
AT	1	23.8	502	10.5	593.5	11.7	1,228	7.6	1,146	21.0	508.5
	2	24.0	500.5	7.1	596	6.8	1,225	13.9	32	24.5	508.5
	3	17.2	528.5	8.5	579	9.8	720.5	17.9	30.5	25.6	507.5
	4	15.3	1,148	8.4	712	8.9	718.5	15.6	25.5	18.6	507
	5	19.2	1,141	8.2	709.5	8.9	717.5	20.9	35.5	16.9	632.5
	6	19.6	1,139	6.9	736.5	16.2	736.5	17.5	30.5	17.6	640.5
	7	22.6	1,154	12.5	837.5	21.0	739.5	14.6	121	23.1	360
	8	14.1	213.5	9.0	835.5	21.2	667.5	11.8	121	22.4	396.5
	9	13.7	214	9.0	277.5	23.9	761.5	15.2	121	19.6	113
	10	14.3	215	10.4	277	21.9	668	11.7	1,185	18.9	157
	…										

In Table 4, there was a trend in the FT block where latencies moved toward contraction onset by 23–35%. A similar trend was seen in the ET block where latencies moved toward contraction onset by 13–23%. While for AT, four out of the five participants showed latencies increasing (moving away from contraction onset) by 25–71%. Subject S112 was an outlier where they showed unclear latency trends – it was flat for FT, while it increased for ET. Also, S112 exhibited an apparent decrease from task 10–19, while tasks 2–9 showed uncharacteristically short MRP times (all for successful MRPs). Alternatively, for the failed MRPs there were no clear significant shifts in latency that appeared with task repetition. For example, rather than a gradual change in latency, the failed tasks exhibit more prominent shifts – indicating higher variability. For example, the average standard deviation of latencies for successful tasks was 296.7 ms, while for failed tasks it was 338.8 ms.

### 3.5 Slow-wave MRP characteristics

Figure 3 depicts MRP plots for S108 during the exercise blocks. The dashed red lines of best fit show MRP peak locations movement, while dashed black lines of best fit show the trough locations movement. Each task MRP (attempt #) is shown on the y-axis, while successful (left) and failed (right) tasks are shown in separate plots. From this, the previously discussed variability in the failed attempts is more clearly depicted by the peak markers in red, while the successful plots show a more uniform trend across the experiment. More specifically from Table 5, S108 depicts a change in latency of −12.2 ms per task away from contraction onset for the successful MRPs and 1.5 ms per task towards contraction onset for failed attempts. Similarly, S112 also displayed shifts away from contraction onset in successful tasks and shifts towards contraction onset for failed tasks (−11.4 ms/task, 11.2 ms/task, respectively). S102 also displayed a similar shift away from onset in successful tasks, but this trend was continued in the failed attempts at a lesser degree (−5.3 ms/task, 1.7 ms/task, respectively). Lastly, S104 was found to have latency shifts towards contraction onset for both successful tasks and failed tasks (8.5 ms/task, 27.9 ms/task, respectively), with S114 also showing a shift towards contraction onset for successful tasks (5.4 ms/task, fail attempts N/A). In successful tasks, there is a pattern that shows a gradual shift in latency between MRP peak away from contraction onset, which is not evident in failed tasks for three of five participants. Conversely, the remaining two participants displayed different directions of change.

**Table 5:** MRP average peak latency Δ.

Sub	Success (ms/task)	Fail (ms/task)
S102	−5.3	−1.7
S104	8.5	27.9
S108	−12.2	1.5
S112	−11.4	11.2
S114	5.4	N/A

From the distributions of the MRP features extracted from Figure 3, it was found that the peak amplitude of the MRPs occurred on average between 726 ms and 467 ms prior to contraction onset. Additionally, the trough (or local minimum) following the peaks occurred on average occurred between 228 ms prior to onset and 61 ms after contraction onset, and with lower variability than the MRP peak latency. There was a pre-peak trough that commonly occurred prior to the MRP peak, which was found to occur on average between 1,278 ms and 1,040 ms prior to motion onset.

**Figure 5:**
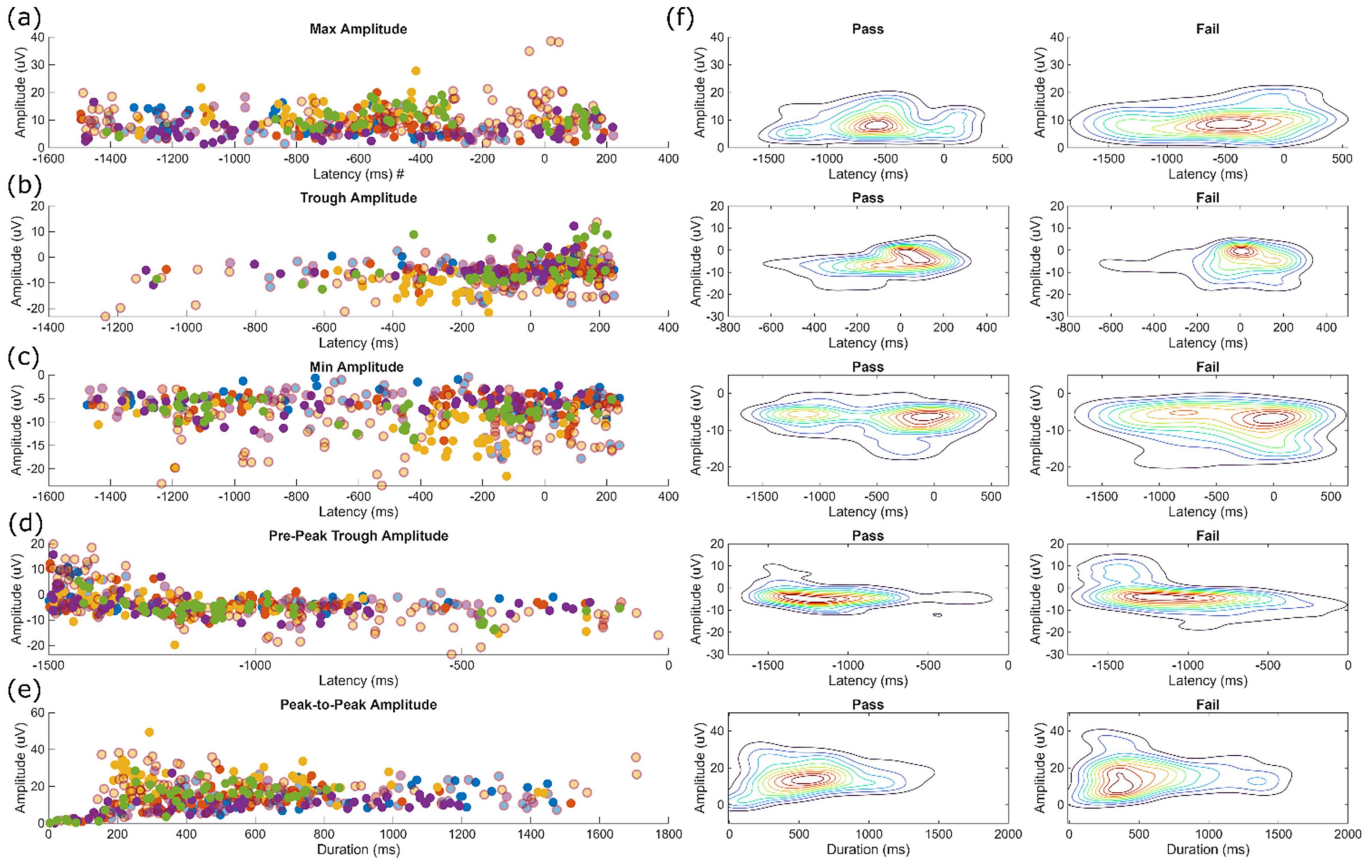
MRP feature scatterplots with marker colors for each subject color coded (Sub102, Sub104, Sub108, Sub112, Sub114). **(A–E)** show distributions for 5 features vs. latency **(F)** contour plots for each distribution. Successes and fails are shown with bold color matching outlines. The contour maps depict the kernel density estimation for success/fail data distribution.

The MRP features from Table 6 were plotted as distributions relative to the latency across all participants in Figures 5A–D with color coded markers (Sub102, Sub104, Sub108, Sub112, Sub114) while failed attempts were depicted with pronounced outlines in the respective colors. These distributions were used to create contour plots, Figure 5F, to compare distributions across successes/fails. Contour plots use latency as the x-axis, where t = 0 is the motion onset time, while the y-axis is the corresponding feature amplitude from Figures 5A–E in microvolts. It was found that the maximum amplitude (Figure 5F top) contour had a centroid at -565 ms and 8.1 μV for the successes, and -492 ms and 8.5 μV for the failures. The centroid for minimum amplitude (Figure 5F center) feature was initially centered at −67.7 ms and −6.21 μV for successful attempts and shifts to −18.2 ms and −6.82 μV in failed attempts. Similarly, the Peak-to-trough vs. duration feature (Figure 5F bottom) was centered at 579 ms and 13.6 μV for successes and 355 ms and 8.79 μV for the fails. While the other two features showed negligible centroid differences between successes and fails.

**Table 6:** Random subject LMM statistics.

Feature	*β* (trials)	*p*-values (trials)	FDR *p-*values	*β* (Conds)	*p*-values (Conds)	FDR *p*-values	*β* (inter)	*p*-values (inter)	FDR *p*-values
Max Amp. (μV)	0.076	9.46E-08	9.46E-07	2.090	0.006	0.029	−0.085	1.41E-04	0.001
Max Lat. (ms)	1.837	0.203	0.226	69.554	0.391	0.627	−2.797	0.234	0.391
Trough Amp. (μV)	0.031	0.067	0.115	−1.444	0.113	0.334	0.028	0.294	0.399
Trough Lat. (ms)	−2.315	0.005	0.026	−33.705	0.468	0.627	0.912	0.498	0.553
Min Amp. (μV)	−0.021	0.069	0.115	−0.931	0.133	0.334	0.047	0.010	0.033
Min Lat. (ms)	−3.887	0.011	0.037	49.363	0.565	0.627	−3.752	0.131	0.274
Pre-P Trough Amp. (μV)	−0.033	0.065	0.115	−0.901	0.363	0.627	0.017	0.557	0.557
Pre-P Trough Lat. (ms)	0.894	0.412	0.412	20.626	0.736	0.736	−2.646	0.137	0.274
P–P Amp. (μV)	0.038	0.144	0.180	3.739	0.004	0.029	−0.116	0.003	0.013
P–P Dur. (ms)	−2.138	0.102	0.145	−42.172	0.513	0.627	1.929	0.319	0.399

To determine the significance of these results between task performance, trial number and the relationship between trial and performance, mixed effect models were applied to the data sets. The initial random intercept model results are shown in Table 6. The fixed effects Max Amplitude (*β* = 0.076, FDR-adjusted *p* <0.001), Trough Latency (*β* = −2.315, FDR-adjusted *p* = 0.026) and Minimum Latency (*β* = −3.887, FDR-adjusted *p* = 0.037) showed significant changes between trials after FDR correction. More specifically, Max Amplitude increased by ~0.076 μV between failed attempts, Similarly, Trough latency moved away from contraction onset by ~2.3 ms per failed attempt, and Minimum Latency shifted away from contraction onset by ~3.9 ms per failed attempt. From the task performance effect, the Maximum Amplitude (*β* = 2.09, FDR-adjusted *p* = 0.029) and P–P Amplitude (*β* = 3.739, FDR-adjusted *p* = 0.029) were found to be significant between successful and failed attempts at baseline (e.g., Success 1 vs. Fail 1). In these cases, the Maximum Amplitude was ~2.09 μV greater in successful attempts than in failed attempts. Similarly, the P–P amplitude was greater by ~3.739 μV in successful attempts than in failed attempts. Lastly, there were significant differences relative to task performance for Maximum Amplitude (*β* = −0.085, FDR-adjusted *p* = 0.0014), Minimum Amplitude (*β* = 0.047, FDR-adjusted *p* = 0.0329) and P–P Amplitude (*β* = −0.116, FDR-adjusted *p* = 0.0128). Specifically, the Maximum Amplitude decreased by 0.009 μV per successful attempt when compared to failed attempts. Moreover, the P–P amplitude also changed at a slower rate in successful attempts than in failed attempts (−0.078 μV/attempt). Conversely the Minimum amplitude changed at a greater rate in successful attempts than in failed attempts (0.026 μV/attempt). From this, the Max Amplitude was shown to have significance across all three effects throughout the experiment relative to task performance. In the evaluation of the task performance, the successful amplitudes for both Max Amp. and P–P Amp were greater than that of the failed attempt at baseline and changed at a slower rate than failed attempts.

To identify the effect of inter-subject variation, random slope models were applied to the model to each feature and random likelihood ratio test (*p* < 0.05) were used to compare the fits to the original random subject model. The models of best fit results can be found in Table 7 and the random effect results found in Table 8. It was found that five of the features were best modeled with the random *performance|sub* slope, Max. Amplitude, Trough Amplitude, Trough Latency, Min. Amplitude, and P–P Amplitude. With Max. Amplitude (*β* = 0.094, FDR-adjusted *p* < <0.001), Trough Latency (*β* = −2.238, FDR-adjusted *p* = 0.016), Min Amplitude (*β* = −0.033, FDR-adjusted *p* = 0.012), and P–P Amplitude (*β* = 0.066, FDR-adjusted *p* = 0.016) showing significant differences between trials. With no features showing significant differences between task performance, and Max. Amplitude (*β* = −0.097, FDR-adjusted *p* <0.001), Min. Amplitude (*β* = 0.061, FDR-adjusted *p* = 0.002), and P–P Amplitude (*β* = −0.145, FDR-adjusted *p* = 0.001) showed significant differences between task performance between attempts. Alternatively, the random *attempt|sub* slope only fit two features better than the random subject model for the Max. Latency and Min. Latency. For these two features there were no significant differences found for these features. While Max. Latency was initially found to have significant differences between task effects relative to task performance (*β* = −6.830, *p* = 0.014), this finding did not survive FDR correction (FDR-adjusted *p* = 0.072).

**Table 7:** Random subject effect results.

Feature	Sub_Std (95% CI)	Res_Std (95% CI)
Max Amp.	1.809 (0.94, 3.50)	4.197 (3.93, 4.48)
Max Lat.	1.022E-13 NaN, NaN	460.3 (431.1, 491.4)
Trough Amp	1.762 (0.886, 3.51)	5.073 (4.75, 5.42)
Trough Lat	0 (NaN, NaN)	263.6 (246.9, 281.5)
Min Amp	1.633 (0.855, 3.12)	3.448 (3.23, 3.68)
Min Lat.	1.08E-13 NaN, NaN	486.5 (455.7, 519.4)
Pre-P Trough Amp.	1.25E-15 (NaN, NaN)	5.621 (5.27, 6.00)
Pre-P Trough Lat.	7.72E-14 (NaN, NaN)	347.8 (325.8, 371.4)
P–P Amp.	3.223 (1.68, 6.18)	6.6219 (6.17, 7.11)
P–P Dur.	90.2 (42.1, 193.3)	335.7 (312.8, 360.2)

**Table 8:** Best fit LMM statistics.

Feature	Model	*β* (trials)	*p*-values (trials)	FDR *p*-values	*β* (conds)	*p*-values (conds)	FDR*p*-values	*β* (inter)	*p*-values (inter)	FDR*p*-values
Max Amp.	1 + conds|sub *p = 0.0025*	0.094	4.19E-10	4.19E-09	2.272	0.063	0.316	−0.097	1.76E-05	1.76E-04
Max Lat.*	1 + trials|sub *p = 0.0025*	4.760	0.099	0.216	86.834	0.288	0.565	−6.830	0.014	0.072
Trough Amp	1 + conds|sub *p = 0.0194*	0.051	0.189	0.236	−0.944	0.325	0.745	0.001	0.980	0.980
Trough Lat*	1 + conds|sub *p = 0.0559*	−2.238	0.006	0.016	−36.241	0.480	0.798	1.045	0.434	0.543
Min Amp*	1 + conds|sub *p = 0.0071*	−0.033	0.002	0.012	−1.132	0.130	0.433	0.061	0.001	0.002
Min Lat.	1 + trials|sub *p = 0.0004*	−4.058	0.291	0.324	27.753	0.759	0.759	−3.811	0.198	0.283
Pre-P Trough Amp.	1|sub *p = ns*	−0.033	0.065	0.115	−0.901	0.363	0.627	0.017	0.557	0.557
Pre-P Trough Lat.	1|sub *p = ns*	0.894	0.412	0.412	20.626	0.736	0.736	−2.646	0.137	0.274
P–P Amp.*	1 + conds|sub *p = 0.0026*	0.066	0.008	0.016	3.986	0.012	0.119	−0.145	1.09E-04	0.001
P–P Dur.	1|sub *p = ns*	−2.138	0.102	0.145	−42.172	0.513	0.627	1.929	0.319	0.399

^*^Large random effect variance characteristics (see Table 9).

## 4 Discussion

In this study, we explored the cortical activation as defined through MRPs during a repetitive wrist flexion and extension motor task. We explored task performance (passing vs. failing) how its representation changes in these motor-related potentials (MRPs) prior to motion onset. Similarly, we measured the effects of performance using the peak amplitude and latency of the MRPs during pre-motor activity and execution. We hypothesized that the changes in MRPs due to task repetition can be measured through peak and latency characteristics over time. We showed that there was a clear change in MRP features with respect to task performance. For example, there was a shift in peak latency of about 133 ms away from contraction onset in failed attempts when compared to successful attempts. Additionally, we found that there was a 17% decrease in peak-to-peak amplitude in the failed MRPs when compared to successful attempts. This suggests that there was a greater trough following the peaks in the successful MRPs than in the fails, suggesting a change in the overall shape of the pre-motor signal. Furthermore, in the analysis of the slow-wave MRPs, it was found that their characteristics such as the Maximum Amplitude, Minimum Latency, and Trough Latency showed significant differences between trials, as well as Maximum Amplitude, Minimum Amplitude and Peak-to-Peak Amplitude which had significant differences between successful and failed tasks over the span of the experiment (FDR-adjusted *p* < 0.05). Due to the heterogeneity of the experiment’s duration, the use of learning here is representative of short-term adaptation. For the analysis of long-term motor learning a multi-session study is needed.

### 4.1 Task performance

Table 1 demonstrated that the subjects had a high degree of variance when performing the motor task. Regardless, we were able to demonstrate that changes in MRP occurred with task repetition, suggesting that MRPs change with motor learning (Jochumsen et al., 2017). Specifically, we found that the number of failed tasks decreased with repetition in most subjects. For example, the number of failures decreased across blocks – the FT block (performed first) had more failures across study participants than the ET (performed second). In fact, it was commonly observed that when starting the experiment, subjects would produce inverse control of the cursor on the screen (flexion goes up, extension goes down). Regardless, the change in performance over time suggests that the subjects experienced motor adaptation while using the HMI over time, since their overall task performance improved. Notably, the AT block saw an increase in failed tasks when compared to the ET, however, this could have occurred because it was a more complex task, using multiple muscles and moving the cursor in different directions. Regardless, the difficulty using the HMI provided a novel task to most participants for repetition-based motor learning.

### 4.2 Task performance effect on ERP

In the evaluation of task performance’s effect on MRP, it was shown that there was a difference when comparing the successes and failures. Specifically, the change in MRP was more gradual in consecutive successful tasks, and more abrupt across consecutive failures. We hypothesize that this may be caused due to learning having different effects if a task is completed successful or not. For example, the decrease in MRP latency with successful tasks may indicate that learning mechanisms optimize using gradual tuning mechanisms in the motor controller – this is supported by earlier research (Turnham et al., 2012). Conversely, failed tasks saw more abrupt changes in MRP which may be caused in the motor controller “searching” for different solutions using random variation (Hossner et al., 2016).

In our data, we additionally observed that the successful task MRP peaks were followed by greater troughs (minima), causing an increase in Peak-to-Peak amplitudes when compared to fail MRPs. This is confirmed by the average maximum peak amplitude, latency and peak-to peak value across all channels, for all subjects (grand average). Specifically, the average maximum amplitude for the MRPs for the successful tasks was 24% closer to motion onset than failed tasks, and an average Peak-to-Peak amplitude decreased 17% in failed tasks. It indicates that the latency was closer to onset in successful tasks while the peak-to-peak amplitude also increased (i.e., difference in execution). This may suggest faster motor preparation time for faster or learned motions(Ariani and Diedrichsen, 2019).

Although the amplitude of the peaks did not appear to change significantly across successful or failed tasks, other changes to MRP morphology between successes and failures suggest a difference in cortical activation. For example, a study by Jankelowitz and Colebatch explored the effect of induced weakness on MRPs and found that they became less bi-phasic (i.e., decreased peak-to-peak amplitude) when weakness increased. Alternatively, when an individual was at their strongest, the MRP peak was more prominent(Jankelowitz and Colebatch, 2002). Although we do not explore weakness, similar assumptions can be made for tasks that are more proficiently performed. In our study, we note some similar findings where we observed a 17% decrease in peak-to-peak amplitude for the fail MRPs when compared to the success MRPs.

A study conducted by Cunningham, et al., showed similar results when comparing healthy participants to patients with Parkinson’s Disease (Cunnington et al., 1997). They noted a change in the MRPs, possibly due to weaker muscle activation. An additional study conducted by Slobounov and Ray suggests that the MRP peak occurs earlier when prioritizing fast action rather than accuracy (Slobounov and Ray, 1998). We see similar shifts in our peak in failed attempts, suggesting that subject failure may be due to individuals attempting to perform the task more quickly.

There were two main trends visible in our data. The first was that the maximum peaks occurred closer to contraction onset in the frontal channels (F3, Fz, F4) and with a greater amplitude than over the M1 (C3, C4) and parietal lobes (P3, P4) for Success MRPs. Conversely, for the failed attempts, MRP peak amplitudes decreased across all channels (3–14%) and the latency for the frontal channels shifted away from contraction onset. The second visible trend was in the variance of the MRP peak latencies. With the exclusion of channels C4 (15ms) and P3 (8ms), all other channels showed a difference in standard deviation of over 160 ms between successes and failures. This suggests that the successful MRPs were more consistent across the group than in the failed attempts.

### 4.3 Task repetition and slow-wave MRPs

Repetition plays a significant role in motor learning which induces transient and long-lasting neuroplastic changes to the corticospinal system (Classen et al., 1998; Carey et al., 2002, 2005). As such, we evaluated cortical MRPs during the repetitive motor control task to identify changes that occur during our experiment. We extracted amplitude and latency data from the MRPs and found that a shift toward contraction onset may represent faster cortical processing. There were some differences across the group, however. For example, S102, S108, and S112 had trends where the peak location moved away from contraction onset while the remaining 2 participants displayed more consistent shifts towards contraction onset across the three experimental blocks. We can speculate that these differences could be related to their motor execution performance patterns. This can be seen where S102, S108, and S112 all had lower task performance success rates of 52, 37 and 56%, respectively, along with longer time per test (~13 s). On the other hand, S104 and S114 were considered the two strongest performers with task success rates of 78 and 98%, respectively. This may suggest that the participants who experienced more failures, could have relied more on sensor-based motor control adjustments, while individuals who performed the task more successfully relied on trained internal models generated through task repetition. This agrees with earlier research that found sensory-dependent motor control has longer latencies than forward model-based control (Le Naour et al., 2023).

To further evaluate these trends, a median filter was applied to explore the slow-wave MRPs (Figure 3). It was found that the most significant features were the Maximum Peak Amplitude vs. Latency, the Trough Amplitude vs. Latency, Minimum Amplitude vs. Latency and the Peak-to-Peak Amplitude vs. Duration. Specifically, after FDR correction we found that the feature amplitude was a strong determinant between successful or failed task execution. Meaning that the amplitude differences in the MRP are related to outcome. Similarly, we found that the shift in MRP progresses with repetition. Since successful task completion is related to this shift, and the amount of shift is dependent on repetition count, the feature appears to be an indication of motor learning for the task. Notably, these features are associated with patterns found in other studies related to MRPs in both healthy populations and populations with neuromuscular diseases. For example, after the MRP peak we show that there is a steep negative slope followed by the greatest minimum (trough). This could be associated with the early BP/RP seen in traditional MRPs which are related to motor preparation. Additionally, the peak-to-peak amplitude and duration are associated with that of the late BP/NS or the shift from motor preparation into execution (Colebatch, 2007). Further studies by Cunningham, et al., as well as Staines, et al., have both shown that when giving a visual cue prior to motion there is a pre-motor positivity that is seen is visuomotor tasks (Cunnington et al., 1997; Staines et al., 2002). This possibly suggests that the positive peaks extracted from the MRPs could be related to the motor control response to visual cues.

This was further supported by Figure 5, which showed the feature distribution centroid shift from 565 ms to 492 ms (13%) for the maximum peaks. In terms of the amplitude, the pre-peak trough, trough, and peak-to-peak amplitude, there was an overall decrease from successes to fails. One explanation for the decreased amplitude seen in failures could be due to weaker force production, as it is tied to MRP amplitude (Oda and Moritani, 1996; Siemionow et al., 2000). This possibly suggests that failed task attempts may be caused by inadequate forces generated to reach and hold the target. Finally, the failed attempts showed peak-to-peak duration decrease in 3 of the 4 participants, which was shown by a shift in the distribution centroid from 579 ms to 355 ms (38%). The decreased duration suggests an overall change in MRP shape between the successes and fails. This is supported by earlier studies of fatigue, which found that MRPs for eccentric movements showed increased activation and increased area (longer duration) than in concentric movements, since eccentric movements require greater cortical preparation (i.e., more processing time) (Fang et al., 2001). Similarly, the decrease in duration of the peak-to-peak change could suggest that there was less cortical processing prior to the failed attempts.

Through the linear mixed-effect models (Tables 6–9) we were able to show the inter-trial variation (change between repetition), inter-subject variation and overall differences that occurred between successful and failed task MRPs. Recall, the initial random subject model had significant differences between the task performance effect for the Maximum Amplitude (*β* = 2.09, FDR-adjusted *p* = 0.0288) and P–P Amplitude (*β* = 3.739, FDR-adjusted *p* = 0.029). For successful attempts, the Max. Amp. and P–P Amp. are greater than that of failed attempts. Interestingly, although the |amplitude| was found to be greater in successful attempts than in failed attempts, they decreased over time in successful attempts but increased in failed attempts. This may suggest differences in processing as part of the learning mechanism. Interestingly, since there appears to be an inverse relationship between the change in amplitude and task performance, it could suggest different cortical modulation relative to task performance. To further support this, in successful task amplitudes, their gradual decrease with repetition could result from decreased cortical activations required to execute the task or could be a result of short-term adaptation in subjects (Wright et al., 2012). While for failed attempts, the increase in amplitude over time could suggest an increased intentionality between movements that increase cortical activation (Aliakbaryhosseinabadi et al., 2017; Suwandjieff et al., 2026). These results are also supported by the work of Hill et al. (2021) where, both positive and negative feedback models were found to shift MRP peaks further from movement onset (Hill et al., 2021). Thus, if successful attempts are related to positive feedback, and failed attempts are related to negative feedback, the measured shift away from contraction onset could be part of earlier pre-motor planning to achieve the best outcomes. This includes the possibility of different cortical modulation and its dependency on the successful outcomes of a task.

**Table 9:** Best fit LMM random effect results.

Feature	Model	Sub_Std (95% CI)	x|sub_Corr (95% CI)	x_Std (95%CI)	Res_Std (95% CI)
Max Amp.	1 + conds|sub	1.56 (0.71, 3.43)	−0.33 (−0.898, 0.652)	2.06 (0.903, 4.69)	4.11 (3.85, 4.39)
Max Lat.*	1 + trials|sub	17.1 (0.36, 813.7)	−1 (NaN, NaN)	4.7 (4.43, 5.05)	448.6 (419.99, 479.1)
Trough Amp	1 + conds|sub	1.201 (0.40, 3.58)	0.244 (−0.852, 0.943)	1.667 (0.697, 3.99)	5.008 (4.69, 5.35)
Trough Lat*	1 + conds|sub	5.6 (0.029, 1094.6)	1 (NaN, NaN)	51.54 (48.26, 55.03)	260.1 (243.6, 277.8)
Min Amp*	1 + conds|sub	1.142 (0.73, 1.78)	1 (NaN, NaN)	0.98 (0.630, 1.52)	3.406 (3.19, 3.64)
Min Lat.	1 + trials|sub	225.5 (106.1, 479.5)	−0.98 (−0.999, −0.086)	7.1 (3.19, 15.81)	471.2 (440.8, 503.6)
Pre-P Trough Amp.*	1|sub	1.25E-15 (NaN, NaN)	–	–	5.621 (5.27, 6.00)
Pre-P Trough Lat.*	1|sub	7.72E-14 (NaN, NaN)	–	–	347.8 (325.78, 371.37)
P–P Amp.*	1 + conds|sub	2.003 (1.05, 3.82)	1 (NaN, NaN)	2.195 (1.14, 4.19)	6.513 (6.07, 6.99)
P–P Dur.	1|sub	90.3 (42.1, 193.3)	–	–	335.7 (312.8, 360.2)

Lastly, from the application of random slope models, LMMs of best fit were found for each feature (Table 8). These random slope models took inter-subject differences into consideration while modeling the effects of inter-trial changes, task performance, and trial changes with respect to task performance. While improving model fit, the *cond|sub* random slope model found significant differences between failed attempts for the Trough Latency, Max. Amplitude, Min. Amplitude, and P–P amplitude. Suggesting that for these features, the data was best modeled when task performance for each subject was taken into consideration. If we recall the trends found from Figure 3 and Table 5, three of five participants displayed shifts in peak latency away from contraction onset in successful attempts that were not replicated in failed attempts, while the remaining participants displayed shifts towards contraction onset for both successful and failed attempts. By using this random slope model, these differences are considered when determining the significance differences for the features. From the models there were slight differences that were found in the beta values for the features. For example, for the Max. Amplitude, the random subject model had a *β* = 0.076 μV between failed attempts and *β* = −0.085 μV for successful attempts relative to failed attempts, while the random slope model found these values to be *β* = 0.094 μV and *β* = −0.097 μV, respectively. Thus, by including these individual conditions in the model, there was a slight increase in feature amplitude. A similar increase was found for both the Min. Amplitude and P–P amplitude as well. Further emphasizing the differences in amplitude that occurred between successful and fail MRP features, as well as how they changed over the course of the experiment.

## 5 Conclusion

This study demonstrates that MRPs change with task repetition and performance outcomes. More specifically we identify key features (Max. Amp, Min. Amp, P–P Amp.) that display significant differences between performances outcomes as well as overtime. Suggesting that within the subjects there existed different learning/adaptation profiles dependent on task performance. Based on our findings, we hypothesize that short-term motor learning manifests by changing the morphology of MRP waveforms, such that the latency and amplitudes can be affected. Using MRPs to assess human task performance, we propose that trends in repetitive task execution can be used to measure the learning rate of the individual. As such, this may therefore have utility in some translational applications, such as predicting patient outcomes during occupational therapy, or understanding pathological impacts on motor control over time. It should be noted these are preliminary findings from a small size and interpreted as such, due to the large inter-subject variability. For this reason, these hypotheses will be explored further in future studies.

### 5.1 Limitations

We would like to note some limitations of this study. First, there was heterogeneity across our study population when performing the task. Specifically, S114 only failed 1 task, while S105 failed the tasks more than 350 times. For example, S114 needed 10 min to complete the task, while S105 needed more than an hour. Due to this heterogeneity, this study should be repeated on a larger scale to better understand population-level trends in MRP changes due to task repetition. Additionally, due to this difference in time of task, the interpretation of learning may differ between participants, for example S114 who had the least time on task is unlikely to experience permanent neuroplastic changes but did display shifts in cortical activity over time suggesting short-term adaptation/learning occurred. Furthermore, due to the small sample size, feature significance was affected by the defined random effects. For example, when evaluating the pre-peak trough, both the amplitude and latency were best modeled with the original subject random effect model. Conversely, when modeling the minimum amplitude feature, the amplitude was best modeled with the random *performance|sub* slope model and the latency was best modeled with the random *attempt|sub* slope model. An additional limitation was that failed attempts were classified using a threshold of the cursor-target position. While this method was shown to produce significance differences between MRP features, it is not certain that each failure was a result of failed planning/executions. Another perspective is the learning curve in using the HMI, since some failures could be a result of learning to work with the device. Further analysis into cursor dynamics should be explored to clarify this.

## Data Availability

The raw data supporting the conclusions of this article will be made available by the authors, without undue reservation.
